# *Ageratum houstonianum* Extract and Agerarin Promote Hair Growth via MAPK/AP-1 Axis-Dependent Upregulation of SCUBE3 in Human Dermal Papilla Cells

**DOI:** 10.3390/ijms27083679

**Published:** 2026-04-20

**Authors:** Yongjin Kim, Euitaek Jung, Gyungmin Cho, Yena Choi, Soon Young Shin

**Affiliations:** 1 Department of Biological Sciences, College of Lifesciences, Konkuk University, Seoul 05029, Republic of Korea; jikjin12@konkuk.ac.kr (Y.K.); mylife4sci@konkuk.ac.kr (E.J.); yyenaa421@konkuk.ac.kr (Y.C.); 2 Skin Science Lab, AgeraMedi, Inc., Seoul 05029, Republic of Korea; n_eee0@ageramedi.com

**Keywords:** activator protein-1, agerarin, *Ageratum houstonianum*, dermal papilla, hair follicles, mitogen-activated protein kinase, signal peptide-CUB-EGF-like domain-containing protein 3

## Abstract

Dermal papilla (DP) cells orchestrate hair follicle growth and cycling by secreting signaling molecules that stimulate follicular epithelial stem cells. The signal peptide CUB-EGF-like domain-containing protein 3 (SCUBE3) was recently identified as a potent anagen stimulator secreted by DP cells. *Ageratum houstonianum* ethanolic extract (AHE) and its active constituent agerarin exhibit anti-inflammatory properties; however, their effects on hair follicle growth remain unclear. This study aimed to investigate the effects of AHE and agerarin on SCUBE3 expression in primary human DP cells and to elucidate the underlying molecular signaling pathway. Cell viability was assessed by measuring cell confluency. Ex vivo hair growth was analyzed using organ cultures of human hair follicles. Gene and protein expression were determined using reverse transcription-PCR, immunoblot analysis, immunofluorescent staining, tyramide signal amplification-based multiplex immunohistochemistry, and gene promoter-reporter assay in primary human follicle DP cells. In a hair follicle organ culture model, both AHE and agerarin increased the population of the anagen phase and promoted hair shaft elongation. AHE and agerarin significantly upregulated SCUBE3 expression at both the mRNA and protein levels. Mechanistically, AHE and agerarin induced activator protein-1 (AP-1) expression by activating mitogen-activated protein kinase signaling pathways, thereby increasing *SCUBE3* gene promoter activity. AHE and agerarin promoted hair follicle growth by upregulating SCUBE3 expression via activation of the MAPK–AP-1 signaling axis. In conclusion, AHE and agerarin may serve as potential therapeutic agents for the prevention and treatment of alopecia (hair loss).

## 1. Introduction

Hair follicles function as dynamic mini-organs that undergo cyclic phases of growth (anagen), apoptosis-driven regression (catagen), and relative quiescence (telogen) throughout life [1]. A complex network of signaling molecules tightly regulates the transition between these phases, which are exchanged between mesenchymal dermal papilla (DP) cells at the base of the follicle and surrounding epithelial cells, including hair follicle stem cells [1]. DP cells are considered the primary regulatory hub in the hair follicle niche because their secreted factors are critical for inducing the anagen phase and maintaining hair growth [2]. The disruption of DP-derived signaling can lead to various hair loss disorders, including androgenetic alopecia. However, the exact range of DP factors that directly promote hair growth remains unclear.

Signal peptide-CUB-EGF-like domain-containing protein 3 (SCUBE3) is a member of the SCUBE family of extracellular signaling modulators [3]. Recent studies have identified SCUBE3 as a DP-derived niche factor that plays a critical role in hair follicle activation and growth [4,5,6]. These studies demonstrated that SCUBE3 was selectively expressed in DP fibroblasts during the anagen phase but was absent in telogen follicles, and it promoted the proliferation of hair follicle stem cells, thereby initiating anagen and supporting robust hair growth. Furthermore, exogenous SCUBE3 has been shown to induce anagen re-entry in both murine and human scalp hair follicles, establishing SCUBE3 as a potent regulator of hair follicle regeneration and a promising therapeutic target for alopecia.

Despite these advances, the upstream regulatory mechanisms controlling SCUBE3 expression within the hair follicle niche remain largely undefined. Previous studies have primarily focused on the functional role of SCUBE3 as a downstream effector, with limited understanding of how extracellular stimuli or pharmacological agents modulate its transcription in DP cells. In particular, the signaling pathways and transcription factors that directly regulate SCUBE3 expression have not been clearly elucidated. Therefore, identifying the molecular mechanisms that govern SCUBE3 expression is essential for developing targeted therapeutic strategies for hair loss.

Natural compounds are important sources of therapeutic agents, and there is increasing interest in plant-derived molecules for dermatological applications, including hair growth promotion [7,8]. Precocene II (6,7-dimethoxy-2,2-dimethyl-2H-chromene), also known as agerarin, is a natural benzofuran derivative isolated from *Ageratum houstonianum* ethanolic extract (AHE) [9]. Although previous studies have reported its anti-inflammatory and antimelanogenic effects in the skin [9,10], its role in regulating hair follicle biology and hair growth remains unclear.

The transcription factor activator protein-1 (AP-1) is a key transcription factor that mediates cellular responses to extracellular stimuli, including proliferation, differentiation, and survival [11], and has been implicated in hair follicle regeneration [12]. AP-1 activity is tightly regulated by the mitogen-activated protein kinase (MAPK) signaling pathway [13]. Notably, agerarin modulates MAPK signaling [9], suggesting a potential link between agerarin-mediated MAPK activation and transcriptional regulation of hair growth-associated genes. Based on this rationale, we hypothesized that AHE and agerarin induce SCUBE3 expression through activation of the MAPK-AP-1 signaling axis DP cells. To test this, we investigated the effects of AHE and agerarin on SCUBE3 expression in primary human follicle dermal papilla (HFDP) cells and evaluated their impact on hair growth using an ex vivo human hair follicle model. Furthermore, we sought to elucidate the underlying transcriptional mechanism linking MAPK signaling to SCUBE3 regulation.

## 2. Results

### 2.1. Examining the Cytotoxic Levels of AHE and Agerarin in Human Follicle Dermal Papilla Cells

**Figure 1:**
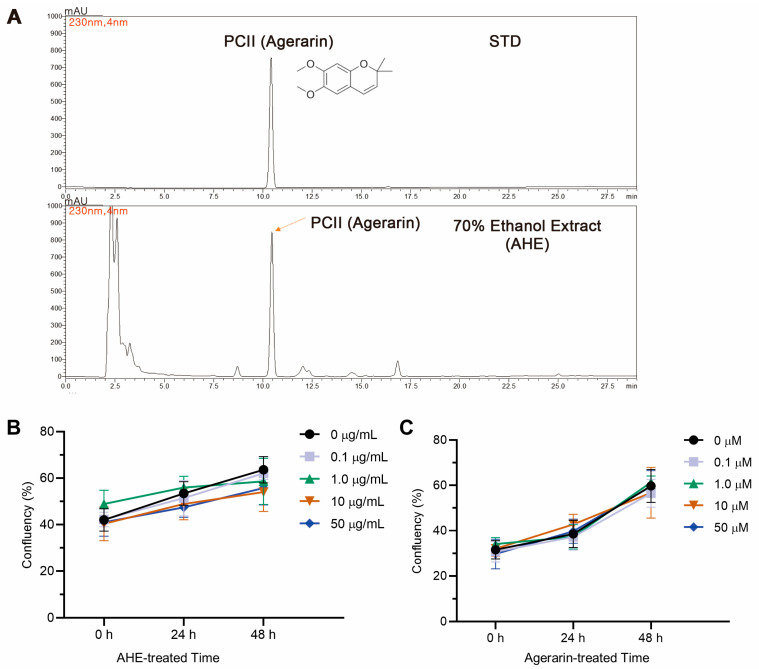
Effect of AHE and agerarin on the viability of human follicle dermal papilla (HFDP) cells. (**A**) Analytical high-performance liquid chromatography (HPLC) profiles of authentic standard (STD) precocene II (PCII, agerarin; **top panel**) and 70% *Ageratum houstonianum* ethanolic extract (AHE; **bottom panel**) using a Shimadzu SIL-40 HPLC-DAD system with a Watchers C18 column (4.6 × 250 mm, 5 μm). PCII STD (1 mg/mL) and AHE (10 mg/mL) were injected with a volume of 10 μL at a flow rate of 0.7 mL/min and detected at 230 nm. (**B**,**C**) Cell viability of primary HFDP cells treated with indicated concentrations of AHE (**B**) and agerarin (**C**) for 24 and 48 h, assessed by measuring cell confluency.

The presence of the active constituent precocene II, known as agerarin, in the AHE was verified through high-performance liquid chromatography (HPLC) analysis conducted by OPTBIO Inc (Figure 1A). Non-cytotoxic concentrations of AHE and agerarin were subsequently tested in primary HFDP cells. For up to 48 h, treatment with AHE (Figure 1B) and agerarin (Figure 1C) at concentrations up to 50 μg/mL and 50 μM, respectively, did not significantly alter cell viability compared with that of the vehicle control. Further investigation was conducted at 50 μg/mL AHE and 50 μM agerarin, exhibiting the highest non-toxic concentrations tested.

### 2.2. AHE and Agerarin Promote Hair Growth of Human Scalp Hair Follicles in an Ex Vivo Organ Culture

**Figure 2:**
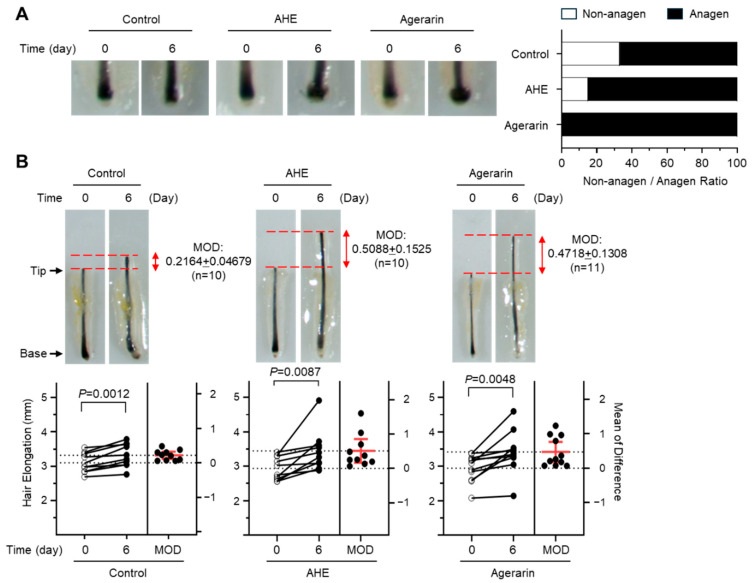
Effect of AHE and agerarin on hair growth in ex vivo organ culture of human scalp hair follicles. Hair follicles were incubated with 50 μg/mL AHE or 50 μM agerarin for 6 days in phenol-free William’s E medium. (**A**) The anagen and non-anagen phases were determined based on the morphological characteristics of the dermal papilla. (**B**) Hair shaft elongation was quantified by measuring the distance from the base to the distal tip using calibrated digital images. Data are presented as mean ± SEM (n = 10−11). MOD, mean of difference representing the change in hair shaft length (length on Day 6 minus length on Day 0). A paired two-tailed *t*-test was used to determine the *p*-value. Dotted lines represent the 95% confidence interval for the difference between the paired data.

We utilized human scalp hair follicles ex vivo to investigate whether AHE and agerarin influenced hair growth. In the control group, 66.7% of hair follicles were in the growth phase (anagen), whereas the AHE-treated (50 μg/mL) and agerarin-treated (50 μM) groups were 84.6% and 100%, respectively (Figure 2A). After 6 days of ex vivo culture, AHE and agerarin significantly increased hair elongation by 2.35- and 2.18-fold, respectively, on a mean of difference (MOD; D6−D0) basis compared to the control group (Figure 2B). 

### 2.3. AHE and Agerarin Increase SCUBE3 Expression in the DP of Human Scalp Hair Follicles in an Ex Vivo Organ Culture

**Figure 3:**
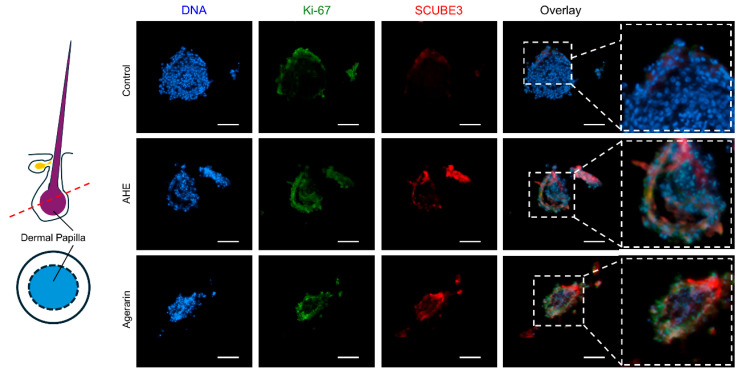
Effect of AHE and agerarin on SCUBE3 expression in ex vivo organ culture of human scalp hair follicles. Hair follicles were incubated with 50 μg/mL AHE or 50 μM agerarin for 6 days in phenol-free William’s E medium. Multiplex immunohistochemistry with tyramide signal amplification in human scalp hair follicles. SCUBE3 and Ki-67 were visualized with ClariTSA 650 (red fluorescence) and ClariTSA 570 (green fluorescence), respectively. Nuclear DNA was visualized with 1 μg/mL Hoechst 33258 (blue fluorescence). Size bar, 100 μm.

SCUBE3 is a DP-derived, pro-anagen signaling molecule that promotes hair follicle growth through activation of the TGF-β/SMAD pathway [5]. Notably, fluorescence immunohistochemical analysis demonstrated strong SCUBE3 immunoreactivity in Ki67-positive proliferating cells in ex vivo-cultured human scalp hair follicles after AHE and agerarin treatment (Figure 3).

### 2.4. AHE and Agerarin Increase SCUBE3 Protein Levels in Primary HFDP Cells

**Figure 4:**
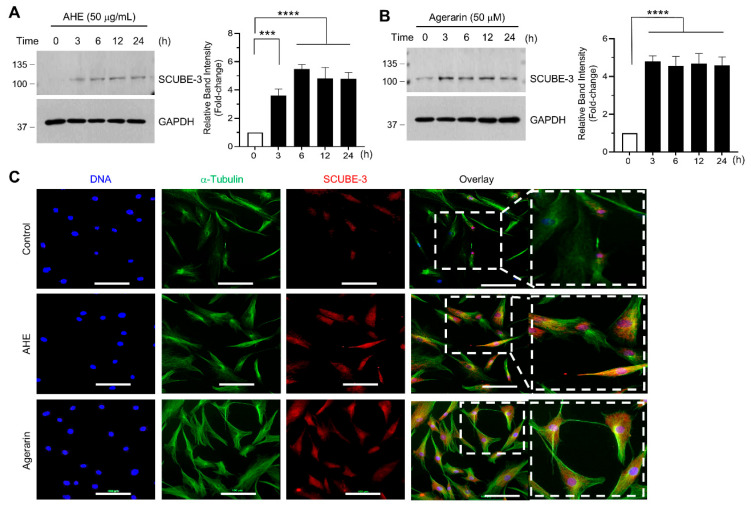
Effect of AHE and agerarin on SCUBE3 protein levels. (**A**,**B**) Immunoblot analysis of SCUBE3 protein levels in primary HFDP cells treated with 50 μg/mL AHE (**A**) or 50 μM agerarin (**B**) for the indicated times. GAPDH was used as a loading control. Relative band intensities were measured using ImageJ 1.53k. Data are presented as mean ± SD (n = 3). *** *p* = 0.001, **** *p* < 0.0001 vs. control using a Dunnett’s multiple comparisons test. (**C**) Primary HFDP cells cultured on coverslips were treated with 50 μg/mL AHE or 50 μM agerarin for 3 h. After fixation and permeabilization, immunofluorescence staining was performed with anti-SCUBE3 primary antibody and Alexa Fluor 647-conjugated secondary antibody (red fluorescence). α-Tubulin was counterstained using anti-tubulin primary and Alexa Fluor 488-conjugated secondary antibodies (green fluorescence). Nuclear DNA was visualized with Hoechst 33258 (blue). The areas in the dashed boxes are magnified in the right panels. Scale bar, 100 μm.

Next, we investigated the mechanisms underlying the AHE- and agerarin-induced SCUBE3 expression in primary HFDP cells. Immunoblot analysis demonstrated that both AHE (Figure 4A) and agerarin (Figure 4B) increased SCUBE3 protein levels within 3 h, which was sustained for up to 24 h. Double immunofluorescence staining further confirmed the enhanced SCUBE3 protein expression (Figure 4C).

### 2.5. AHE and Agerarin Increase SCUBE3 mRNA Levels in Primary HFDP Cells

**Figure 5:**
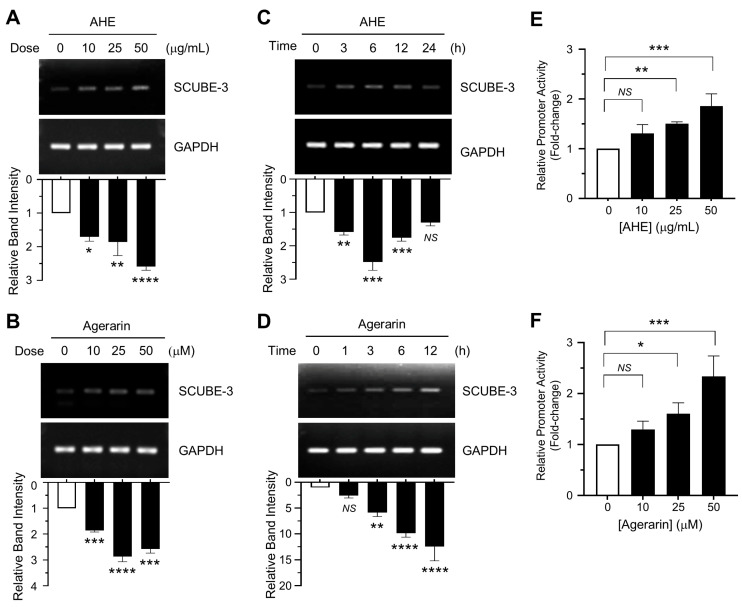
Effect of AHE and agerarin on enhancing *SCUBE3* mRNA expression. (**A**,**B**) Relative *SCUBE3* mRNA levels in primary HFDP cells treated with various concentrations of AHE (**A**) and agerarin (**B**) for 3 h, measured by RT-PCR. * *p* = 0.0115, ** *p* = 0.0036, **** *p* < 0.0001 (**A**); *** *p* = 0.0001, **** *p* < 0.0001 (**B**). (**C**,**D**) Time-course of relative *SCUBE3* mRNA expression in primary HFDP cells treated with 50 μg/mL AHE (**C**) and 50 μM agerarin (**D**). *^NS^ p* = 0.0687, ** *p* = 0.0013, *** *p* = 0.0002 (**C**); *^NS^ p* = 0.4481, ** *p* = 0.0040, **** *p* < 0.0001 (**D**). Relative band intensities were measured using ImageJ (**A**–**D**). (**E**,**F**) Primary HFDP cells were co-transfected with the 100 ng pScube3-Luc(−972/−20) reporter plasmid. After 24 h, the cells were treated with increasing concentrations of AHE (**E**) and agerarin (**F**). Luciferase activities were measured after an additional 12 h. Data are presented as mean ± SD (n = 3)). *^NS^ p* = 0.0930, ** *p* = 0.0092, *** *p* = 0.0003 (**E**); *^NS^ p* = 0.3627, * *p* = 0.0384, *** *p* = 0.0004 (**F**) vs. control by Dunnett’s multiple comparisons test.

Consistent with the protein levels, both AHE (Figure 5A) and agerarin (Figure 5B) led to a significant dose-dependent increase in *SCUBE3* mRNA levels. A time-course experiment using 50 μg/mL AHE (Figure 5C) and 50 μM agerarin (Figure 5D) showed that *SCUBE3* mRNA expression began to increase within 3 h and remained elevated for at least 24 h. To address whether the *SCUBE3* mRNA induction occurs at the gene promoter level, we generated a promoter-reporter construct containing the 5′-flanking region of *SCUBE3*, designated pScube3-Luc(−972/−20), and transfected it into primary HFDP cells. Treatment with AHE (Figure 5E) and agerarin (Figure 5F) significantly increased *SCUBE3* promoter-reporter activity in a dose-dependent manner. 

### 2.6. AP-1 Is Involved in Agerarin-Induced SCUBE3 Expression

**Figure 6:**
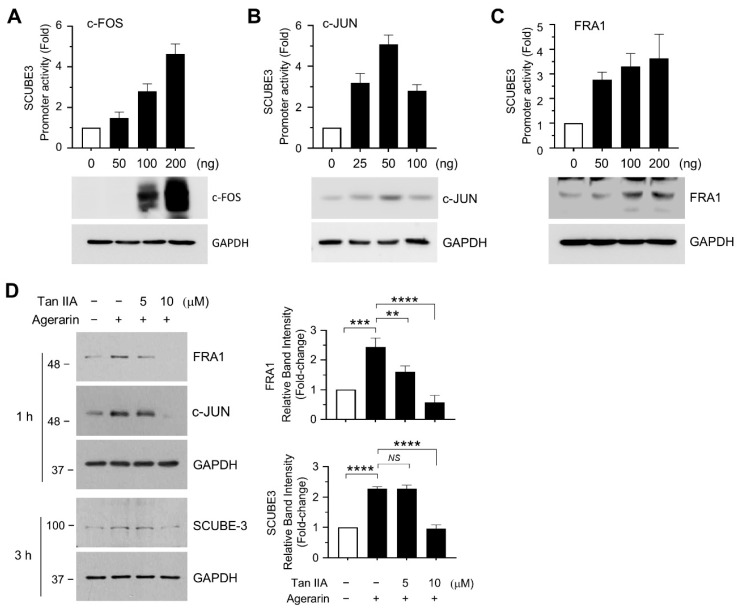
Role of AP-1 in agerarin-induced SCUBE3 expression. (**A**–**C**) Primary HPDP cells were co-transfected with the pScube3-Luc(−972/−20) reporter and increasing concentrations of the c-FOS (**A**), c-JUN (**B**), or FRA1 (**C**) expression plasmids. After 48 h, cells were harvested, and luciferase activity was measured (**top graph**). Transfected proteins were determined by immunoblotting (**bottom panels**). Bars represent means ± SD (n = 3). (**D**) Primary HFDP cells were pretreated with 5 or 10 μM Tanshinone (Tan)-IIA for 30 min, then stimulated with 50 μM agerarin. After 1 or 3 h, whole-cell lysates were subjected to immunoblot analysis using antibodies against FRA1, c-JUN, and SCUBE3. GAPDH was used as a loading control. Relative band intensities were measured using ImageJ (**right graphs**). Data are presented as mean ± SD (n = 3). ** *p* = 0.0038, *** *p* = 0.00038, **** *p* < 0.0001 (FRA1); *^NS^ p* > 0.9999, **** *p* < 0.0001 (SCUBE3) by Dunnett’s multiple comparisons test.

The AP-1 transcription factor complex plays a critical role in hair follicle development and regeneration [12]. To investigate its involvement in regulating *SCUBE3* transcription, we analyzed the 5′-flanking regulatory region of the *SCUBE3* gene in the human genome assembly (GRCh38/hg38), spanning from −972 to −20 bp relative to the transcription start site (TSC; Supplementary Figure S1). Analysis using the JASPAR database (https://jaspar.genereg.net/ (accessed on 21 July 2025) predicted multiple AP-1-binding motif clusters within the upstream region from −972 to −20 bp (Supplementary Figure S2). To evaluate the contribution of AP-1 to *SCUBE3* transcriptional regulation, primary HFDP cells were co-transfected with pScube3-Luc(−972/−20) promoter-reporter construct and an expression plasmid encoding the AP-1 subunit (c-FOS, c-JUN, or FRA1) to assess AP-1-dependent activation of the *SCUBE3* promoter. Transient expression of c-FOS, c-JUN, or FRA1 (Figure 6A–C) significantly enhanced *SCUBE3* promoter-reporter activity in a plasmid concentration-dependent manner. To further determine the role of AP-1 in SCUBE3 expression, we used an AP-1 modulator. Tanshinone IIA (Tan-IIA), a Chinese herbal compound derived from *Salvia miltiorrhiza*, suppresses AP-1 subunit expression by inhibiting MAPK signaling [14]. Pretreatment of primary HFDP cells with 5 or 10 μM Tan-IIA for 60 min led to a significant reduction in the agerarin-induced AP-1 subunit FRA-1 and SCUBE3 protein levels (Figure 6D).

### 2.7. MAPK Pathways Contribute to AHE- and Agerarin-Induced SCUBE3 Expression

**Figure 7:**
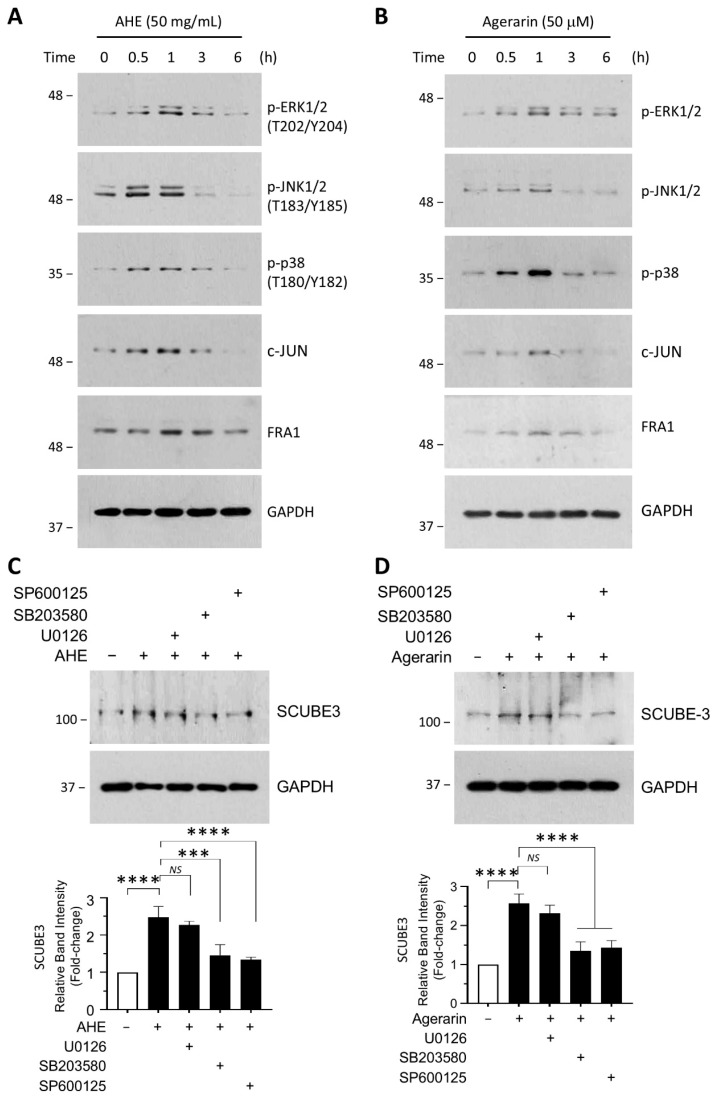
Role of MAPK pathways in AHE- and agerarin-induced SCUBE3 expression. (**A**,**B**) Primary HFDP cells were treated with 50 μg/mL AHE (**A**) and 50 μM agerarin (**B**) for the indicated times. Whole-cell lysates were subjected to immunoblotting with antibodies against p-ERK1/2, p-JNK1/2, p-p38, and FRA1. (**C**,**D**) Primary HFDP cells were pretreated with 10 μM U0126, 10 μM SB203580, or 20 μM SP600125 for 30 min before stimulation with 50 μg/mL AHE (**C**) or 50 μM agerarin (**D**). Whole-cell lysates were subjected to immunoblotting with antibodies against SCUBE3. GAPDH served as a loading control. Relative band intensities were measured using ImageJ. Data are presented as mean ± SD (n = 3). *^NS^ p* = 0.5146, *** *p* = 0.0002, **** *p* < 0.0001 (**C**); *^NS^ p* = 0.3639, **** *p* < 0.0001 (**D**) by Dunnett’s multiple comparisons test.

AP-1 is a well-established downstream effector of MAPK signaling pathways [15,16]. To determine whether AHE and agerarin modulate MAPK activity, we examined the phosphorylation status of key MAPK subfamilies in primary HFDPCs. Treatment with AHE (Figure 7A) and agerarin (Figure 7B) induced a rapid and transient increase in the phosphorylated forms of ERK, JNK, and p38, detectable as early as 0.5 h and peaking at approximately 1 h, followed by a gradual decline toward baseline levels by 6 h. This temporal pattern was accompanied by increased expression of the AP-1 subunit FRA1. These findings are consistent with activation of MAPK signaling and its downstream AP-1 axis in response to AHE and agerarin.

To address the potential involvement of MAPK signaling in AHE- and agerarin-induced SCUBE3 expression, the effects of pharmacological inhibitors were evaluated by immunoblot analysis. Pretreatment with the p38 kinase inhibitor SB203580 and the JNK inhibitor SP600125 significantly attenuated SCUBE3 protein accumulation induced by 50 μg/mL AHE (Figure 7C) and 50 μM agerarin (Figure 7D). In contrast, pretreatment with the MAPK kinase (MEK) inhibitor U0126, which acts upstream of ERK, had minimal effect on SCUBE3 expression. These findings suggest that the JNK and p38 MAPK pathways play a crucial role in mediating SCUBE3 induction by AHE and agerarin, whereas the ERK pathway appears to be dispensable for this process.

## 3. Discussion

Our findings show that AHE and its active constituent, agerarin, enhance SCUBE3 expression by activating the MAPK–AP-1 signaling axis, suggesting that AHE or agerarin could potentially serve as therapeutic agents for alleviating and treating alopecia (hair loss).

SCUBE3 has recently emerged as a key DP-derived signaling molecule that promotes hair follicle activation and anagen induction [5,6]. Previous studies demonstrated that SCUBE3 acts as a paracrine factor that stimulates hair follicle stem cells and facilitates hair regeneration [5]. However, these studies primarily focused on the functional role of SCUBE3, providing limited insight into the upstream signaling pathways that regulate its expression.

The upregulation of SCUBE3 in DP cells has important biological implications for hair follicle regeneration. As a secreted niche factor, SCUBE3 is positioned to function as a key mediator of epithelial–mesenchymal interactions within the hair follicle microenvironment. Increased SCUBE3 expression in DP cells may enhance the activation of hair follicle stem cells, promote the transition from telogen to anagen, and support sustained hair shaft growth.

Our ex vivo human hair follicle culture results support this concept, as treatment with AHE and agerarin increased hair shaft elongation and the proportion of follicles in the anagen phase. These findings suggest that modulation of SCUBE3 expression in DP cells may represent a mechanism by which extracellular stimuli influence hair growth dynamics.

In parallel, the MAPK signaling cascade and its downstream transcription factor AP-1 have been widely implicated in hair follicle biology, including DP cell proliferation, differentiation, and response to external stimuli [17]. Activation of ERK, JNK, and p38 MAPKs has been shown to regulate hair growth-associated genes and contribute to anagen progression. In this context, our findings provide new evidence that MAPK signaling, particularly the p38 and JNK pathways, regulates SCUBE3 expression through AP-1-dependent transcriptional mechanisms. This expands the current understanding of SCUBE3 biology by linking extracellular signaling pathways to its transcriptional regulation in DP cells.

The DP is a distinct mesenchymal structure located at the base of the hair-follicle bulb [18]. During the onset of anagen, the DP releases active signals, including Wnts [19,20], FGF7 [21], SHH [12], and SCUBE3 [5], that stimulate bulge stem cells, amplify matrix cells, and promote the proliferation and differentiation into the lineages of the hair shaft and inner root sheath [22]. SCUBE3 is a potent DP cell-derived mitogen for hair follicle stem cells and represents a new avenue for developing hair growth-promoting therapies [5]. The primary goal of this study was to investigate whether natural AHE and its bioactive constituent, agerarin, could modulate SCUBE3 expression in human DP cells and to elucidate the underlying molecular mechanisms. Our findings show that both AHE and agerarin effectively upregulated SCUBE3 expression at the transcriptional level in primary HFDP cells.

Furthermore, we demonstrated that AHE- and agerarin-induced upregulation of SCUBE3 expression was mediated through the activation of the MAPK–AP-1 signaling axis. The effects of AHE and agerarin on hair growth were validated in ex vivo human hair follicle organ cultures. It is worth noting this result because increasing endogenous SCUBE3 production in DP cells promotes the natural physiological process of anagen induction [5]. By increasing SCUBE3 production, agerarin may amplify crucial paracrine signals from the DP niche that are required to awaken dormant hair follicle stem cells and drive the follicle into the anagen phase, potentially promoting hair growth and preventing alopecia.

The AP-1 is a transcription factor composed of a homo- or heterodimer of proteins from the JUN, FOS, ATF, and MAF families. AP-1 regulates gene expression in response to multiple extracellular signals, such as growth factors, cytokines, stress, and differentiation signals [23]. The current understanding of the role of AP-1 in hair growth highlights its context-dependent, stage-specific function during the hair follicle cycle. The growth phase of hair follicles is tightly regulated by signaling pathways, including Wnt/β-catenin, TGF-β, and MAPK cascades [24]. AP-1 acts as a downstream nuclear effector of these pathways that regulate proliferation and differentiation in keratinocytes and hair follicle cells, consistent with a role for AP-1 in mediating the proliferative transcriptional program in the anagen hair matrix [12,17].

c-JUN, a core component of AP-1, directly regulates transcription of genes involved in cell cycle progression and represses cell cycle inhibitory programs [25], thereby supporting the role of AP-1 in hair follicle growth. An experimental study conducted in goats showed that *JunB* is highly expressed in DP cells during stages of hair follicle development, and functional assays demonstrated that JunB significantly increased DP cell viability and proliferation [26], underscoring the requirement of AP-1 for follicle development and regeneration.

The MAPK signaling pathway is a highly conserved cascade that transduces extracellular stimuli into cellular responses, including proliferation, differentiation, and survival [11]. In mammals, the MAPK family comprises three well-characterized subfamilies: ERKs, JNKs, and p38 kinases. Activation of these kinases leads to phosphorylation and regulation of numerous downstream targets, including AP-1 transcription factor [13]. The MAPK pathway regulates AP-1 activity at multiple levels, including the induction of FOS, JUN, and FRA1 expression, and the post-translational phosphorylation of AP-1 proteins, which enhances their transcriptional activity [15,16]. Thus, AP-1 acts as a downstream effector of MAPK signaling and plays a crucial role in controlling hair follicle regeneration [12].

Our study provides evidence that AP-1 is essential for agerarin-induced SCUBE3 gene expression, as revealed by a *SCUBE3* promoter-reporter assay and the inhibitory effect of the AP-1 modulator Tan-IIA. AHE and agerarin treatment rapidly induced AP-1 subunits, including c-JUN, c-FOS, and FRA1, in HFDP cells. JNK is the primary kinase responsible for phosphorylating c-JUN at its N-terminal serine residues (Ser63/73), thereby dramatically enhancing its transactivation potential [27]. An increase in the number of AP-1 subunits (c-FOS, c-JUN, and FRA1) likely results in the formation of highly active AP-1 heterodimers that bind to the promoter region of the *SCUBE3* gene to drive its transcription. We observed that AHE and agerarin increased the phosphorylation of ERK, JNK, and p38 kinase. Thus, AHE and agerarin stimulate the *SCUBE3* promoter by activating the MAPK–AP-1 signaling axis. This hypothesis warrants further investigation in future studies using techniques such as in vivo chromatin immunoprecipitation.

The Wnt/β-catenin signaling pathway is a central regulator of hair follicle development, regeneration, and DP cell function [28]. Activation of β-catenin signaling promotes the transition into the anagen phase and is widely recognized as a key driver of hair growth [19,20,29]. In the present study, we demonstrate that AHE and agerarin induce SCUBE3 expression by activating the MAPK/AP-1 signaling axis. Although our data support a primary role for the MAPK/AP-1/SCUBE3 pathway, it remains possible that Wnt/β-catenin signaling also contributes to the hair growth-promoting effects of AHE and agerarin. Although direct evidence linking SCUBE3 to the Wnt/β-catenin signaling pathway remains limited, extensive crosstalk between growth factor-mediated signaling pathways, including TGF-β and Wnt/β-catenin, has been well documented [30]. Notably, SCUBE3 has been reported to function as a secreted factor that enhances hair follicle regeneration, potentially through modulation of epithelial–mesenchymal signaling networks, which may intersect with Wnt signaling [5]. Furthermore, Wnt signaling induces SCUBE3 expression in ovarian cancer cells [31]. Therefore, AHE- and agerarin-induced hair growth may involve coordinated activation of multiple pathways, including MAPK/AP-1/SCUBE3 and Wnt/β-catenin signaling. Further studies are required to determine whether Wnt signaling is activated independently or downstream of SCUBE3 in this context.

The discovery of a plant-derived compound that enhances the endogenous anagen-promoting pathway provides new mechanistic insights and a potential therapeutic strategy for alopecia and other hair growth disorders. Current Food and Drug Administration-approved treatments for alopecia, such as minoxidil and finasteride, have limitations, including variable efficacy and potential side effects [32]. Agerarin, a natural compound with anti-inflammatory activity in the skin, promotes hair growth by enhancing endogenous anagen-activating signals, suggesting a novel mechanism-based strategy for the treatment of alopecia. By stimulating SCUBE3 expression in DP cells, agerarin may reinforce DP-derived pro-anagen signaling that supports follicular growth. This targeted mechanism suggests that agerarin or AHE could serve as a safe and effective alternative or complementary therapeutic option for various forms of hair loss.

Despite these findings, several limitations should be acknowledged. First, although this study observed that AHE- and agerarin induced rapid, transient increases in phospho-ERK, -JNK, and -p38, the corresponding contributions of changes in total ERK, JNK, and p38 protein levels were not comprehensively quantified. Therefore, these findings should be interpreted as indicative of MAPK pathway activation rather than definitive evidence of phosphorylation-specific regulation alone. Second, our study is primarily based on in vitro experiments using primary human DP cells and ex vivo human hair follicle organ culture models. Although these systems are widely used and physiologically relevant, they do not fully recapitulate the complexity of the in vivo hair follicle niche. Third, while our data support a role for MAPK/AP-1 signaling in regulating SCUBE3 expression, direct evidence of transcription factor binding, such as chromatin immunoprecipitation (ChIP) analysis, was not included in this study. Finally, the absence of in vivo validation limits the ability to fully assess the therapeutic potential and systemic effects of AHE and agerarin. Future studies using animal models will be necessary to confirm the role of the MAPK–AP-1–SCUBE3 axis in hair growth under physiological conditions.

Although our findings suggest that AHE and agerarin may have potential as modulators of hair growth, these results should be interpreted with caution. The current study provides mechanistic insight into SCUBE3 regulation rather than definitive evidence of clinical efficacy. Further preclinical and clinical studies are required to evaluate the safety, efficacy, and pharmacokinetic properties of these compounds before their potential therapeutic or cosmetic applications can be established.

## 4. Materials and Methods

### 4.1. Cell Culture and Reagents

Primary HFDP cells isolated from human dermis of the scalp were purchased from PromoCell (Heidelberg, Germany) and cultured in Follicle Dermal Papilla Cell Growth medium (PromoCell) supplemented with 10% fetal bovine serum, 100 U/mL penicillin, and 100 μg/mL streptomycin at 37 °C in a humidified atmosphere of 5% CO_2_. Cells from passages three to six were used for all experiments. AHE was obtained from OPTBIO Inc (Gangneung-si, Gangwon-do, Republic of Korea). Agerarin (>98% purity) was purchased from Sigma-Aldrich (St. Louis, MO, USA) and dissolved in dimethyl sulfoxide to prepare a stock solution. The MEK1/2 inhibitor U0126, c-Jun N-terminal kinases (JNK) inhibitor SP600125, and p38 inhibitor SB203580 were purchased from Cell Signaling Technology (Danvers, MA, USA). ClariTSA Fluorophore Kit 650 (Cat. No. 7527) and ClariTSA Fluorophore Kit 570 (Cat. No. 7526) were obtained from Tocris (Avonmouth, Bristol, UK).

### 4.2. Antibodies

Antibody against SCUBE3 was obtained from Abcam (Cat. No. ab189955; Cambridge, UK). Phospho-ERK1/2 (Thr202/Tyr204; Cat. No. #9101), phospho-JNK (Thr183/Tyr185; Cat. No. #9251), phospho-p38 (Thr180/Tyr182; Cat. No. #9211S), c-JUN (Cat. No. #9165S) antibodies were from Cell Signaling Technology (Danvers, MA, USA). GAPDH (Cat. No. sc-32233) and FRA1 (Cat. No. sc-48424) antibodies were from Santa Cruz Biotechnology (Dallas, TX, USA). Ki-67 antibody (Cat. No. 27309-1-AP) was from Proteintech (Rosemont, IL, USA). Alexa Fluor 647-conjugated goat anti-rabbit IgG (H+L) (red fluorescence; Cat. No. A21244) was from Invitrogen (Carlsbad, CA, USA), and Alexa Fluor 488-conjugated affinipure Goat-Rat IgG (H+L) (green fluorescence; Cat. No. 164822) was obtained from Jackson ImmunoResearch (West Grove, PA, USA).

### 4.3. Cell Viability Assay

HFDP cells were seeded at a density of 5 × 10^4^ cells/well in 12-well plates. The following day was designated as Day 0. On day 1, cells were treated with different concentrations of AHE (0–50 μg/mL) or agerarin (0–50 μM). Phase-contrast images were acquired at 24 and 48 h using an Eclipse TS100 microscope (Nikon, Tokyo, Japan). For viability assessment, the cell-covered area (confluency) was quantified from the acquired images using ImageJ 1.53k (NIH, Bethesda, MD, USA; https://jaspar.genereg.net/, accessed on 21 July 2025), and values were normalized to the day 0 measurement for each well to calculate the relative confluency over time. The experiments were performed in triplicate and repeated three times independently.

### 4.4. Ex Vivo Hair Growth Assay

Human hair follicles were isolated from scalp skin and cultured ex vivo at Epi Biotch Co. (Incheon, Republic of Korea) as previously described [33], with minor modifications. Briefly, individual follicles were microdissected to remove surrounding components, including adipose tissues, and cultured in 48-well plates containing Williams’ E medium (Gibco^®^, Thermo Fisher Scientific, UK) supplemented with L-glutamine, insulin, hydrocortisone, and antibiotics. Cultures were maintained at 37 °C in a humidified atmosphere with 5% CO_2_. Each group used 10−11 hair follicles. AHE (50 μg/mL) or agerarin (50 μM) was added to each well at the start of culture. On day 6 post-treatment, individual hair follicles were assessed for hair cycle stage (anagen vs. non-anagen) based on established morphological criteria for human hair follicle organ culture [34]. Hair shaft elongation was quantified by measuring the distance from the follicle base to the distal tip using calibrated digital images. Subsequently, hair follicles were fixed in paraformaldehyde for immunofluorescence analysis.

### 4.5. Fluorescent Multiplex Immunohistochemistry Staining

Formalin-fixed human scalp hair follicles were embedded in paraffin to generate formalin-fixed paraffin-embedded blocks. Sections (5 µm) were obtained and prepared for staining procedures. Sections were deparaffinized and rehydrated using xylene and graded ethanol. Antigen retrieval was performed by heating the slides for 20 min in Tris-based buffer (pH 9.0) containing 0.05% Tween-20. Endogenous peroxidase activity was quenched by incubation with 3% H2O2 for 30 min at room temperature, followed by permeabilization with 0.3% Triton X-100 in TTBS and blocking with 2% bovine serum albumin (Bovogen, Melbourne, VIC, Australia) and 2% goat serum (Gibco, Waltham, MA, USA) in TTBS. Sections were incubated overnight at 4 °C with primary antibodies against SCUBE3 (1:500) and Ki-67 (1:500). Signals were detected using horseradish peroxidase (HRP)-conjugated secondary antibodies and a tyramide signal amplification (TSA) system, employing a ClariTSA Fluorophore Kit 650 (1:200) for SCUBE3 and a ClariTSA Fluorophore Kit 570 (1:100) for Ki-67. For multiplex staining, TSA detection was performed sequentially with antibody stripping between cycles using a homemade SDS/β-mercaptoethanol-based stripping buffer (60 °C, 30 min). Nuclei were counterstained with 1 μg/mL Hoechst 33258, and autofluorescence was reduced by treatment with NH_4_Cl/CuSO_4_. The slides were mounted in an aqueous medium and imaged using a fluorescence microscope (Eclipse Ti2; Nikon). Fluorescence intensity was quantified using ImageJ software (National Institutes of Health). The experiments were repeated three times independently.

### 4.6. Immunoblot Analysis

Primary HFDP cells were seeded in six-well plates and grown to 80–90% confluence. After treatment with vehicle (DMSO; 0.1% *v*/*v*), AHE or agerarin for the indicated concentrations and times, cells were washed with ice-cold PBS and lysed in lysis buffer (50 mM Tris-HCl; pH 7.4, 150 mM NaCl, 1% NP-40, 0.25% Na-deoxycholate, 1 mM EDTA, 1 mM Na_3_VO_4_, 1 mM NaF, 10 μg/mL leupeptin, and 1 mM phenylmethylsulfonyl fluoride). Protein concentrations were determined using the BCA Protein Assay Kit (Thermo Fisher Scientific, Waltham, MA, USA). Equal amounts of protein (20–30 μg) were separated by 10% SDS-PAGE and transferred to polyvinylidene difluoride membranes (Millipore, Billerica, MA, USA). The membranes were blocked with 5% non-fat milk in Tris-buffered saline with 0.1% Tween 20 (TBST) for 1 h at 25 °C. Membranes were incubated overnight at 4 °C with primary antibodies (SCUBE3, 1:1000; phospho-ERK1/2, 1:4000; phospho-JNK, 1:1000; phospho-p38, 1:1000; c-JUN, 1:2000; FRA1, 1:1000; GAPDH, 1:6000) followed by incubating HRP-conjugated secondary antibodies (1:5000) for 1 h at 25 °C. Protein bands were visualized using an enhanced chemiluminescence detection system (Bio-Rad, Hercules, CA, USA) and quantified using ImageJ software (NIH). The experiments were repeated three times independently.

### 4.7. Fluorescent Immunocytochemistry Staining

Primary HFDP cells cultured on coverslips were left untreated or treated with AHE or agerarin. After 3h, the cells were fixed in 4% paraformaldehyde, permeabilized in 0.1% Triton X-100 and 2% bovine serum albumin, and incubated with primary antibodies against SCUBE3 and α-tubulin (for counter staining) for 2 h, followed by addition of Alexa Fluor 647-conjugated (red fluorescence for SCUBE3) and Alexa Fluor 488-conjugated (green fluorescence for α-tubulin) secondary antibodies for 30 min. Nuclear DNA was stained with Hoechst 333258 (blue fluorescent) for 10 min. Fluorescence images were captured using a fluorescence microscope (Eclipse Ti2; Nikon, Tokyo, Japan). The experiments were repeated three times independently.

### 4.8. Reverse Transcription-PCR (RT-PCR)

Total RNA was isolated using the TRIzol RNA Extraction Kit (Invitrogen, Carlsbad, CA, USA), and cDNA was prepared using the iScript cDNA Synthesis Kit (Bio-Rad, Hercules, CA, USA). RT-PCR was performed as described previously [35]. Total RNA (1 µg) was reverse-transcribed into cDNA using the iScript cDNA Synthesis Kit (Bio-Rad). The thermal cycling conditions were: 95 °C for 3 min, followed by 40 cycles of 95 °C for 10 s and 60 °C for 30 s. The primer sequences were as follows:

- SCUBE3 Forward: 5′-CAG AAC ACC CCG AGG TCA TAC-3′
- SCUBE3 Reverse: 5′-GCC AGG GAT GTT GAC ACA GTC-3′
- GAPDH Forward: 5′-GAA GGT GAA GGT CGG AGT C-3′
- GAPDH Reverse: 5′-GAA GAT GGT GAT GGG ATT TC-3′

The experiments were repeated three times independently.

### 4.9. In Silico Prediction of AP-1-Binding Sites Within the SCUBE3 Gene Regulatory Region

The 5′-flanking region of the *SCUBE3* (−972 to −20 relative to the transcription start site (TSS) was analyzed using the UCSC Genome Browser (GRCh38/hg38) and ENCODE regulatory annotations. Putative transcription factor AP-1-binding motifs were predicted using the JASPAR database (https://jaspar.elixir.no/ (accessed on 21 July 2025).

### 4.10. Generation of SCUBE3 Promoter-Reporter Construct

The 5′-regulatory region of the *SCUBE3* (−972 to −20) was amplified from human genomic DNA by PCR using specific primers as follows:

- forward, −972 F: 5′-CTG ACA GGT ACC GGA GCC TCT TGT ACC TGT CT-3′
- reverse, −20 R: 5′-AGT CTC AGA TCT CTC TCT CGC TCT TTC TCC GT-3′.

The PCR amplicon was inserted into a T&A vector (RBC Bioscience, Taipei County, Taiwan) to generate the TA–SCUBE3 construct. The TA–SCUBE3 plasmid was then digested with *Kpn*I and *Bgl*II to excise the promoter insert, which was subsequently subcloned into the *Kpn*I/*Bgl*II sites of the pGL4.17 luciferase reporter vector (Promega, Madison, WI, USA), yielding pScube3-Luc(−972/−20).

### 4.11. SCUBE3 Promoter-Reporter Assay

Primary HFDP cells were cultured in 12-well plates and transfected with 0.2 µg of the pScube3-Luc(−972/−20) promoter-reporter construct using Lipofectamine 2000 (Invitrogen) according to the manufacturer’s instructions. Where indicated, cells were co-transfected with 100 ng of pScube3-Luc(−972/−20) and increasing amounts (0, 50, 100, and 200 ng) of expression vectors encoding c-FOS (pCDNA3.1zeo/c-Fos), c-JUN (pCDNA3.1zeo/c-Jun), or FRA1 (pCDNA3.1zeo/Fra1). After 48 h, luciferase promoter reporter activity was quantified using a Luciferase Assay System (Promega) and a luminometer (Centro LB960; Berthold Tech, Bad Wildbad, Germany). The relative luciferase activity of untreated cells was set to “1”. The experiments were performed in triplicate and repeated three times independently.

### 4.12. Statistical Analysis

All experiments were performed in triplicate, and the data are presented as the mean ± standard deviation (SD) or standard error of the mean (SEM). Statistical analyses were performed using the GraphPad Prism 8 software (GraphPad Software, San Diego, CA, USA). Comparisons between two groups were performed using a paired two-tailed *t*-test for ex vivo hair elongation measurement, and comparisons among multiple groups were performed using one-way analysis of variance followed by Dunnett’s or Sidak’s multiple-comparison tests. Statistical significance was set at *p* < 0.05.

## 5. Conclusions

This study demonstrates that the natural plant extract AHE and its active constituent agerarin upregulate SCUBE3 expression in human DP cells, at least in part through activation of the MAPK–AP-1 signaling pathway. Consistent with this mechanism, AHE and agerarin promoted hair shaft elongation and increased the proportion of anagen-phase follicles in an ex vivo human hair follicle model.

These findings provide mechanistic insight into the upstream regulation of SCUBE3 and suggest that modulation of the MAPK–AP-1–SCUBE3 axis may contribute to hair growth-associated processes. However, given that the present study is based on in vitro and ex vivo systems, further in vivo studies are required to establish the physiological relevance and potential applicability of these findings.

## Data Availability

The original contributions presented in this study are included in the article/Supplementary Material. Further inquiries can be directed to the corresponding author.

## Supplementary Materials

The following supporting information can be downloaded at: https://www.mdpi.com/article/10.3390/ijms27083679/s1.

## Abbreviations

AP-1	activator protein-1
DP	dermal papilla
ERK	extracellular signal-regulated kinases
JNK	c-Jun N-terminal kinases
HFDP	human follicle dermal papilla
MAPK	mitogen-activated protein kinase
RT-PCR	Reverse transcription-PCR
SCUBE3	signal peptide-CUB-EGF-like domain-containing protein 3
TSA	tyramide signal amplification
TSS	transcription start site
