# Supplementary material for: Ageratum houstonianum Extract and Agerarin Promote Hair Growth via MAPK/AP-1 Axis-Dependent Upregulation of SCUBE3 in Human Dermal Papilla Cells
Source: Int J Mol Sci. 2026 Apr 20;27(8):3679. doi: 10.3390/ijms27083679 (PMC13115932; doi:10.3390/ijms27083679)
Supplement: Supplementary file 1 [file ijms-27-03679-s001.zip › Supplementary_Figure_S1.pdf]

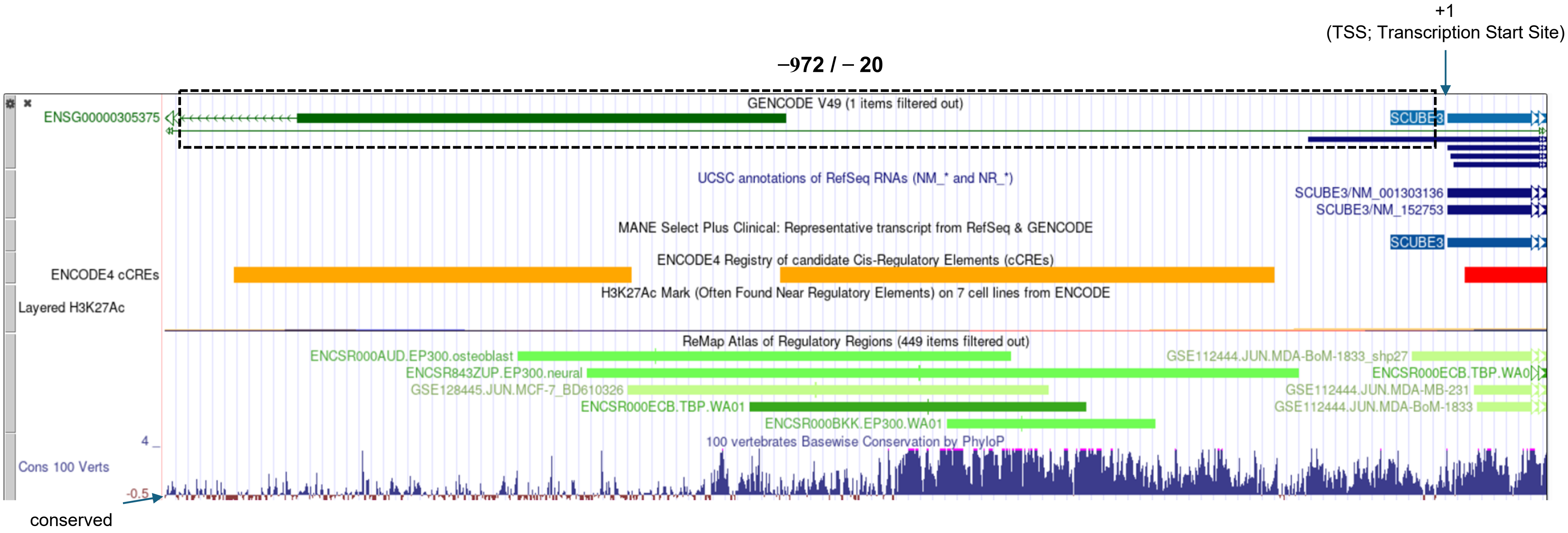

Supp. Figure S1. Graphical representation of the 5' regulatory region of *SCUBE3* from −972 to −20 bp relative to the transcription start site (TSS) in the GRCh38/hg38 human genome assembly. ENCODE regulatory tracks, including candidate *cis*-regulatory elements (cCRE) and H3K27ac marks, as well as vertebrate conserved regions, are indicated.
