# Supplementary material for: Ageratum houstonianum Extract and Agerarin Promote Hair Growth via MAPK/AP-1 Axis-Dependent Upregulation of SCUBE3 in Human Dermal Papilla Cells
Source: Int J Mol Sci. 2026 Apr 20;27(8):3679. doi: 10.3390/ijms27083679 (PMC13115932; doi:10.3390/ijms27083679)
Supplement: Supplementary file 1 [file ijms-27-03679-s001.zip › Supplementary_Figure_S2.pdf]

A

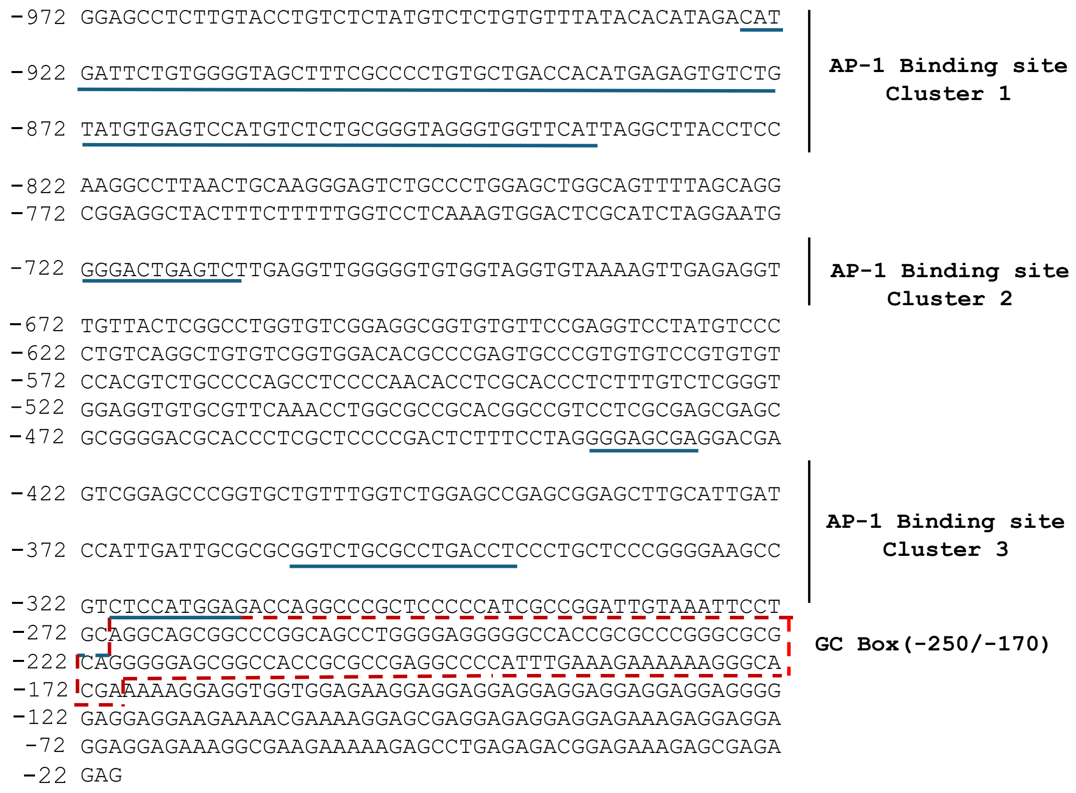

B

Cluster 1

Matrix ID	Transcription factor	Score	Relative score	Sequence ID	Start(TSS)	End(TSS)	(start/end)	Strand	Predicted sequence
MA1142.2	FOSL1::JUND	5.854021	0.8233666	seq1	-896	-889	(-896/-889)	+	ATGATTCT
MA0488.2	JUN	2.944507	0.83163905	seq1	-865	-856	(-865/-856)	-	ATGTGGTCAG
MA0488.2	JUN	2.125108	0.8221876	seq1	-865	-856	(-865/-856)	+	CTGACCACAT
MA0492.2	JUND	1.4976444	0.8227901	seq1	-865	-855	(-865/-855)	-	CATGTGGTCAG
MA0492.2	JUND	0.23701103	0.8091757	seq1	-842	-832	(-842/-832)	+	TGTGAGTCCAT
MA1142.2	FOSL1::JUND	5.289294	0.80730766	seq1	-841	-834	(-841/-834)	+	GTGAGTCC
MA0488.2	JUN	2.525481	0.8268058	seq1	-841	-832	(-841/-832)	+	GTGAGTCCAT
MA1132.2	JUN::JUNB	5.1341286	0.8002313	seq1	-840	-833	(-840/-833)	-	TGGACTCA
MA0489.1	JUN	3.4842417	0.80977046	seq1	-821	-808	(-821/-808)	+	GTAGGGTGGTTCAT
MA1142.2	FOSL1::JUND	6.1927543	0.83299905	seq1	-816	-809	(-816/-809)	+	GTGGTTCA
MA1138.2	FOSL2::JUNB	4.571141	0.8023842	seq1	-816	-808	(-816/-808)	+	GTGGTTCAT
MA0462.3	BATF::JUN	5.7344465	0.8356814	seq1	-840	-834		-	GGACTCA

Cluster 2

Matrix ID	Transcription factor	Score	Relative score	Sequence ID	Start(TSS)	End(TSS)	(start/end)	Strand	Predicted sequence
MA0462.3	BATF::JUN	4.266122	0.802807	seq1	-689	-683		+	TGAGTCT
MA0462.3	BATF::JUN	6.5836024	0.8546932	seq1	-689	-683		-	AGACTCA
MA0489.1	JUN	5.3858175	0.8342402	seq1	-695	-682	(-695/-682)	+	GGGGACTGAGTCTT
MA0099.4	FOS::JUN	6.227143	0.8269158	seq1	-690	-682	(-690/-682)	+	CTGAGTCTT
MA1134.2	FOS::JUNB	6.1211915	0.82946414	seq1	-690	-682	(-690/-682)	+	CTGAGTCTT
MA1141.2	FOS::JUND	6.22433	0.8278806	seq1	-690	-682	(-690/-682)	+	CTGAGTCTT
MA1135.2	FOSB::JUNB	6.086644	0.83127815	seq1	-690	-682	(-690/-682)	-	AAGACTCAG
MA1128.2	FOSL1::JUN	6.2580123	0.8238333	seq1	-690	-682	(-690/-682)	-	AAGACTCAG
MA1137.2	FOSL1::JUNB	7.3771667	0.83540463	seq1	-690	-682	(-690/-682)	-	AAGACTCAG
MA1130.2	FOSL2::JUN	6.1119857	0.82728124	seq1	-690	-682	(-690/-682)	+	CTGAGTCTT
MA1138.2	FOSL2::JUNB	5.822666	0.8257352	seq1	-690	-682	(-690/-682)	-	AAGACTCAG
MA1144.2	FOSL2::JUND	5.9466224	0.8271407	seq1	-690	-682	(-690/-682)	-	AAGACTCAG
MA0490.3	JUNB	6.2654343	0.8163998	seq1	-690	-682	(-690/-682)	-	AAGACTCAG
MA0491.3	JUND	5.671296	0.8092873	seq1	-690	-682	(-690/-682)	-	AAGACTCAG
MA1142.2	FOSL1::JUND	7.462917	0.86911815	seq1	-689	-682	(-689/-682)	-	AAGACTCA
MA1132.2	JUN::JUNB	7.4405775	0.8580908	seq1	-689	-682	(-689/-682)	-	AAGACTCA

Cluster 3

Matrix ID	Transcription factor	Score	Relative score	Sequence ID	Start(TSS)	End(TSS)	(start/end)	Strand	Predicted sequence
MA1132.2	JUN::JUNB	6.2331724	0.8278019	seq1	-441	-434	(-441/-434)	+	GGGACGCA
MA1142.2	FOSL1::JUND	5.416404	0.81092227	seq1	-348	-341	(-348/-341)	-	ATGGATCA
MA0488.2	JUN	0.76188666	0.8064633	seq1	-320	-311	(-320/-311)	-	GGGAGGTCAG
MA0492.2	JUND	2.3114085	0.83157843	seq1	-320	-310	(-320/-310)	-	AGGGAGGTCAG

Supp. Figure S2. Identification of the AP-1-binding site clusters. (**A**) Prediction of AP-1-binding motifs within the 5'-flanking region of the *SCUBE3* gene, spanning from −972 and −20, using the JASPAR database (https://jaspar.elixir.no). Red-coloured dotted box indicates the GC box. (**B**) The positions of potential AP-1-binding motifs in Cluster 1, 2, and 3 regions.
